# P-1007. Head-to-Head Comparison of “Soft Stop” and “Hard Stop” alerts for the Diagnostic Stewardship of Clostridioides difficile PCR testing

**DOI:** 10.1093/ofid/ofaf695.1204

**Published:** 2026-01-11

**Authors:** fnu sonia, Kamil Dzieniszewski, Jamie Palazzo, Vishnu Chaturvedi, Donald S Chen, Marina Keller

**Affiliations:** Westchester Medical center, white plains, NY; Westchester Medical center, white plains, NY; Westchester Medical Center, Valhalla, New York; westchester medical center, Valhalla, New York; Westchester Medical Center, Valhalla, New York; Westchester Medical Center, Valhalla, New York

## Abstract

**Background:**

Clostridioides difficile (Cdiff)-associated diarrhea is a significant nosocomial infection. In the United States, hospitals report every new positive Cdiff test. The financial penalties for high rates of hospital onset tests (i.e. after day 3) incentivize institutions to implement rigorous diagnostic stewardship. Studies have shown that “soft stop” or “hard stop” alerts, limiting options in the order menus, and reflex testing are effective. We conducted a head-to-head comparison of both “soft stop” and “hard stop” alert in the same institution over several years.

**TABLE 1.**
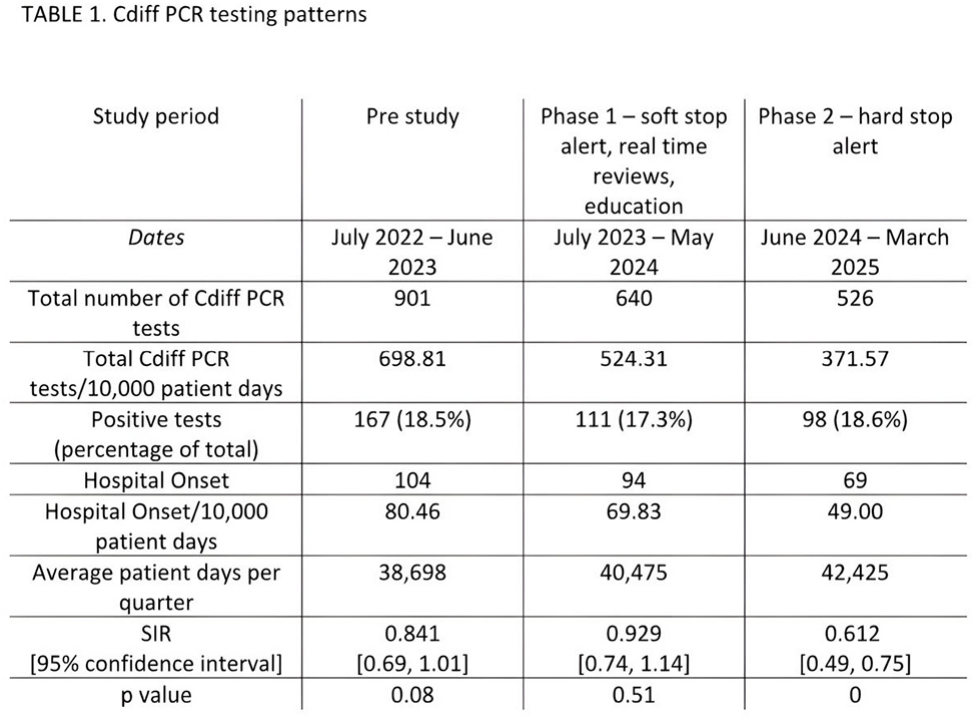
Cdiff PCR testing patterns

**Figure 1:**
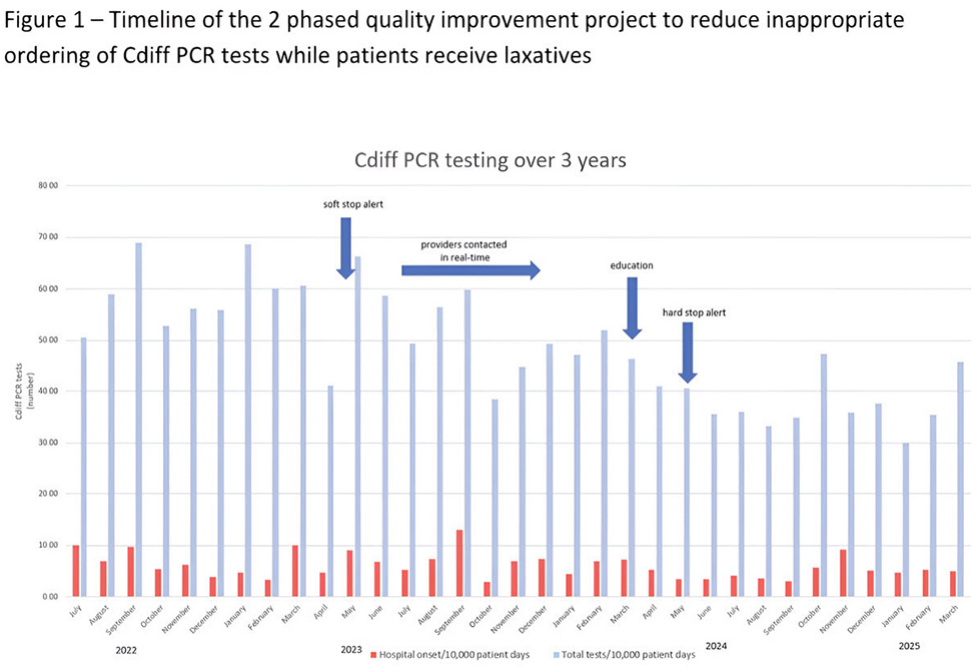
– Timeline of the 2 phased quality improvement project to reduce inappropriate ordering of Cdiff PCR tests while patients receive laxatives

**Methods:**

High rates of positive Cdiff polymerase chain reaction (PCR) tests were noted in a single site, academic center with 652 beds. A real-time review of all positive tests revealed that 35 out of 89 (39.3%) patients had received laxatives within the previous 48 hours. In the first phase of the quality improvement project, a “soft stop” was built into the electronic medical record (EMR) that notified providers not to order a Cdiff PCR test while on laxatives. For 6 months, a physician epidemiologist contacted each provider who ignored the “soft stop” and requested that the test be canceled. Departments with high rates of ordering Cdiff PCR tests were educated about appropriate testing. In phase 2, a “hard stop” was introduced that did not allow any Cdiff PCR tests to be ordered while on laxatives.

**Figure 2:**
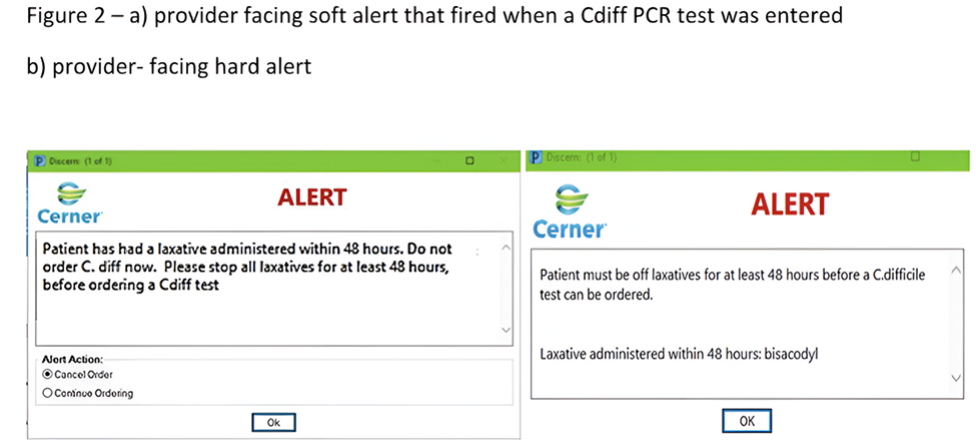
– a) provider facing soft alert that fired when a Cdiff PCR test was entered b) provider- facing hard alert

**Results:**

Overall, Cdiff PCR testing decreased due to the two alerts with a stable positivity rate over the 3 years of the study (figure 1). There was a significant increase in patient days over time which may reflect post pandemic changes in hospitalizations (table 1). There was no difference in the standardized infection rate (SIR) after implementing the “soft stop” (table 1). However, the “hard stop” implementation led to a statistically lower SIR (p=0).

**Conclusion:**

he “soft stop” alert was not effective at lowering the Hospital Onset (HO) rate of Cdiff, but implementation of the “hard stop” alert rapidly brought down the institutional SIR.

**Disclosures:**

All Authors: No reported disclosures

